# Synergistic effects of boron and saponin in mitigating salinity stress to enhance sweet potato growth

**DOI:** 10.1038/s41598-024-63840-z

**Published:** 2024-06-06

**Authors:** Uzma Younis, Subhan Danish, Rahul Datta, Sami Al Obaid, Mohammad Javed Ansari

**Affiliations:** 1 Botany Department, The Islamia University of Bahawalpur, Sub Campus Rahim Yar Khan, Rahim Yar Khan, Punjab Pakistan; 2 Pesticide Quality Control Laboratory, Old Shujabad Road, Multan, 60000 Punjab Pakistan; 3 Department of Geology and Pedology, Faculty of Forestry and Wood Technology, Mendel University in Brno, Zemedelska 1, 61300 Brno, Czech Republic; 4 Department of Botany and Microbiology, College of Science, King Saud University, PO Box-2455, 11451 Riyadh, Saudi Arabia; 5 Department of Botany, Hindu College Moradabad (Mahatma Jyotiba Phule Rohilkhand University Bareilly), Moradabad, India

**Keywords:** Antioxidant activity, Boron, Chlorophyll content, Photosynthetic rate, Saponin, Sweet potato, Plant sciences, Plant stress responses, Abiotic, Salt

## Abstract

Salinity stress significantly hinders plant growth by disrupting osmotic balance and inhibiting nutrient uptake, leading to reduced biomass and stunted development. Using saponin (SAP) and boron (B) can effectively overcome this issue. Boron decreases salinity stress by stabilizing cell walls and membranes, regulating ion balance, activating antioxidant enzymes, and enhancing water uptake. SAP are bioactive compounds that have the potential to alleviate salinity stress by improving nutrient uptake, modulating plant hormone levels, promoting root growth, and stimulating antioxidant activity. That’s why the current study was planned to use a combination of SAP and boron as amendments to mitigate salinity stress in sweet potatoes. Four levels of SAP (0%, 0.1%, 0.15%, and 0.20%) and B (control, 5, 10, and 20 mg/L B) were applied in 4 replications following a completely randomized design. Results illustrated that 0.15% SAP with 20 mg/L B caused significant enhancement in sweet potato vine length (13.12%), vine weight (12.86%), root weight (8.31%), over control under salinity stress. A significant improvement in sweet potato chlorophyll a (9.84%), chlorophyll b (20.20%), total chlorophyll (13.94%), photosynthetic rate (17.69%), transpiration rate (16.03%), and stomatal conductance (17.59%) contrast to control under salinity stress prove the effectiveness of 0.15% SAP + 20 mg/L B treatment. In conclusion, 0.15% SAP + 20 mg/L B is recommended to mitigate salinity stress in sweet potatoes.

## Introduction

Salts naturally occur in soil or are introduced through irrigation or fertilizers^1^. These salts presents a significant environmental challenge, affecting ~ 7% of the world’s land area and increasingly posing a serious issue^2^. Such condition leads to considerable crop loss globally, due osmotic stress, ionic imbalance, and oxidative damage in plants. It also adversely impacts plant physiology, morphology, and various biochemical processes, including water, nutrient uptake, and seed germination^3,4^. Using boron (B) and saponin (SAP) might be an effective amendment when combined to overcome this issue.

Boron stands out among micronutrients as an essential element universally required by all plants^5^. It is a component of cell walls; its biochemical functions remain relatively underexplored. However, its significance in upholding membrane function and supporting various plant metabolic activities is pivotal. It also supports metabolic activities in plants^6^. Similarly, SAP can significantly enhance plant growth by improving nutrient absorption by acting as a natural surfactant^7^. They stimulate root development, aiding in mineral and water uptake. These compounds also foster symbiotic relationships with beneficial soil microbes, promoting nutrient cycling and soil health^8^. However, careful application of SAP is crucial to avoid adverse effects on specific plant species.

The sweet potato (*Ipomoea* *batatas* (L.) Lam.), belonging to the Convolvulaceae family, is a primary staple crop globally, valued for its storage roots and various aerial components utilized in human consumption, animal fodder, and industrial applications^9^. Sweet potatoes are rich in complex carbohydrates, dietary fibre, vitamins, and minerals and offer a nutritious diet^10^. Their adaptability to diverse climates and ability to thrive in marginal lands make them resilient crops, contributing to food security, especially in regions prone to challenging growing conditions^11^. Furthermore, sweet potatoes play a role in enhancing soil fertility due to their ability to reduce soil erosion and improve soil structure, demonstrating their significance not only in food systems but also in sustainable agriculture and environmental conservation^12^. Salinity stress significantly affects the growth, development, and productivity of sweet potatoes.

The current study explores the potential of SAP and B to mitigate the salinity stress in sweet potato plants. The study is novel because of the limited literature regarding the combined use of SAP and B to minimize salinity stress, especially in sweet potato plants. This study covers the knowledge gap for using SAP and B as combined amendments for possible solutions to salinity stress. We hypothesized that applying SAP with B might alleviate salinity stress’s adverse effects on sweet potato plants.

## Material and methods

### Experimental site

A pot experiment was conducted in ResearchSolution, Multan, Punjab, Pakistan, to examine the effect of B and SAP foliar spray on the growth, nutrient concentration and antioxidant activity in sweet potatoes cultivated in salt-affected soil. The experimental site GPS location was 30° 09′ 41.6′′ N 71° 36′ 38.0′′ E. A composite soil sample was made using 6 samples for the pre-experimental soil analysis. The pre-experimental soil characteristics are provided in Table 1.

**Table 1:** Pre-experimental soil and irrigation characteristics.

Soil	Values	References	Irrigation	Values	Reference
pH	8.82	^13^	pH	7.05	^14^
EC*e* (dS/m)	6.94	^15^	EC (µS/cm)	234	
Organic matter (%)	0.50	^16^	CO_3_^–2^ (meq./L)	0.00	
Total nitrogen (%)	0.003	^17^	HCO_3_^–1^ (meq./L)	4.99	
Available phosphorus (µg/g)	6.15	^18^	Cl^−1^ (meq./L)	0.010	
Extractable potassium (µg/g)	109	^19^	Ca^+2^ + Mg^+2^ (meq./L)	1.09	
Extractable sodium (µg/g)	714	^20^	Na^+^ (mg/L)	100	
Texture	Loam	^21^			

###  Collecting and sowing of plant material

We acquired the sweet potato tubers from the local fruit and vegetable market (Desi Shakarkandi, a sensitive cultivar against salinity stress). A single tuber was sown (28 January 2023) in each pot with 15 kg of soil. During the experiment, temperature was maintained at 23 ± 5 °C and relative humidity at 60 ± 5%.

### Saponin and boron

The salt purchased corresponds to SAP product number 47036, batch number BCCK1316, solubility of 1 g in 10 ml water and belongs to the SIGMA brand. The chemical was identified by CAS Number 8047–15-2. For the application of B, borax (Fauji Fertilizers Company Limited = FFC; Pack Size: 3 kg; Salt: Di-Sodium Tetra Borate Decahydrate; Boron: 10.5%) was purchased from a certified fertilizer dealer of Multan. The claim of the product was 10.5% boron.

### Treatment plan and growth conditions

The treatments involved four levels of SAP (0, 0.10, 0.15 and 0.20%) and B (control, 5, 10, and 20 mg/L). The study followed a completely randomized design (CRD) with four replicates. All the foliar solutions were made using deionized sterilized water. SAP was applied in 2 splits, i.e., after 20 days and 40 days of sowing.

### Data collection

Measurements of antioxidants, chlorophyll contents, chlorophyll fluorescence, net photosynthetic rate, stomatal conductance, and transpiration rate were done after a 60 days growth period. However, morphological attributes were noted during final harvesting (after 120 days of sowing).

### Free proline, chlorophyll contents and carotenoids

Initially, healthy fresh leaves from the plant were collected after 50 days of tuber. Carefully, leaves were removed by hand to minimize the chances of minimal physical damage. The collected leaves were stored in a liquid nitrogen container and then transferred to the laboratory to prevent contamination and preserve their integrity. The extraction and analysis of free proline from leaf tissues were then performed following the protocol described by^22^. The absorbance was taken for the complex, made of ninhydrin and proline at 520 nm wavelength, for the final determination of free proline. For chlorophyll and carotenoids, 10 mL of 80% acetone was added to each tube, and extraction was done by taking 1 g of fresh leaf tissues in darkness at room temperature. Subsequently, the absorbance of the samples was measured using a UV–Vis spectrophotometer at 663, 645 and 480 nm wavelengths^23^.

$${\text{Chlorophyll}}\,{\text{ a }}\left( {\frac{{{\text{mg}}}}{{\text{g}}}} \right) = \frac{{\left( {12.7{ } \times {\text{ A}}663} \right){-}{ }\left( {2.69{ } \times {\text{ A}}645} \right) \times {\text{V}}}}{{1000{ } \times {\text{W}}}}$$

$${\text{Chlorophyll}}\,{\text{ b }}\left( {\frac{{{\text{mg}}}}{{\text{g}}}} \right) = \frac{{\left( {22.9{ } \times {\text{ A}}645} \right){-}{ }\left( {4.68{ } \times {\text{ A}}663} \right) \times {\text{V}}}}{{1000{ } \times {\text{W}}}}$$

$${\text{Total }}\,{\text{Chlorophyll }}\left( {\frac{{{\text{mg}}}}{{\text{g}}}} \right) = { }20.2\left( {{\text{OD }}645} \right) + 8.02\left( {{\text{OD }}663} \right) \times {\text{V}}/1000{ }\left( {\text{W}} \right){ }$$

$${\text{Carotenoids }}\left( {\frac{{{\text{mg}}}}{{\text{g}}}} \right){ } = {\text{OD}}480 + 0.114{ }\left( {{\text{OD }}663} \right) - 0.638{ }\left( {{\text{OD }}645} \right)$$

### Chlorophyll fluorescence

The fluorescence emitted from the upper leaf surface (adaxial) was evaluated using a fluorescence monitoring system operating in the pulse amplitude modulation mode. This assessment followed the methodology as detailed by^24^. Furthermore, the photon yield of PSII (˚PSII) during illumination was computed as °PSII = (Fm − F)/Fm after 45 s of continuous light exposure. This duration ensured the attainment of a steady state for accurate measurement^25^.

### Net photosynthetic rate, stomatal conductance, and transpiration rate

The net photosynthetic rate, stomatal conductance, and transpiration rate were assessed using a Portable Photosynthesis System incorporating an infrared gas Analyzer^26^.

### Assay of DPPH radical scavenging activity

A 0.1 ml portion of the diluted sample was mixed with 3.9 ml DPPH solution to initiate the reaction process. The UV-spectrophotometer’s absorbance was measured at 515 nm at one-minute intervals for 180 min. Consequently, a standardized 3 h reaction time was employed for all DPPH assays^27^.

### Assay of ABTS radical scavenging and MDA activity

The method for evaluating ABTS radical-scavenging activity in the hydrophilic fractions followed the protocol outlined by^28^. An ABTS+ solution was prepared by mixing 8 mM of ABTS salt with 3 mM of potassium persulfate in 25 ml of distilled water. This mixture was left in darkness at room temperature for 16 h. To achieve an absorbance between 0.8 and 0.9 at 734 nm, the ABTS+ solution was diluted with 95% ethanol (approximately 600 μl ABTS in 40 ml 95% ethanol). Using a UV- spectrophotometer, absorbance at 734 nm was recorded every minute for 30 min. For measuring malondialdehyde (MDA), a marker of lipid peroxidation, the sample extract was treated with thiobarbituric acid (TBA) to generate a visible complex. The absorbance of this complex was quantified at 532 nm wavelength to determine the concentration of MDA.

### Statistical analysis

Traditional statistical methods were utilized for data analysis, encompassing a two-way ANOVA to assess treatment significance. Paired comparisons underwent the Tukey test, setting significance at *p* ≤ 0.05. OriginPro software^29^ generated cluster plots with convex hulls, hierarchical cluster plots, and Pearson correlations.

### Ethics approval and consent to participate

We all declare that manuscript reporting studies do not involve any human participants, human data, or human tissue. So, it is not applicable. Study protocol must comply with relevant institutional, national, and international guidelines and legislation. Our experiment follows the with relevant institutional, national, and international guidelines and legislation.

## Results

### Vine length, vine weight, and number of leaves

**Figure 1:**
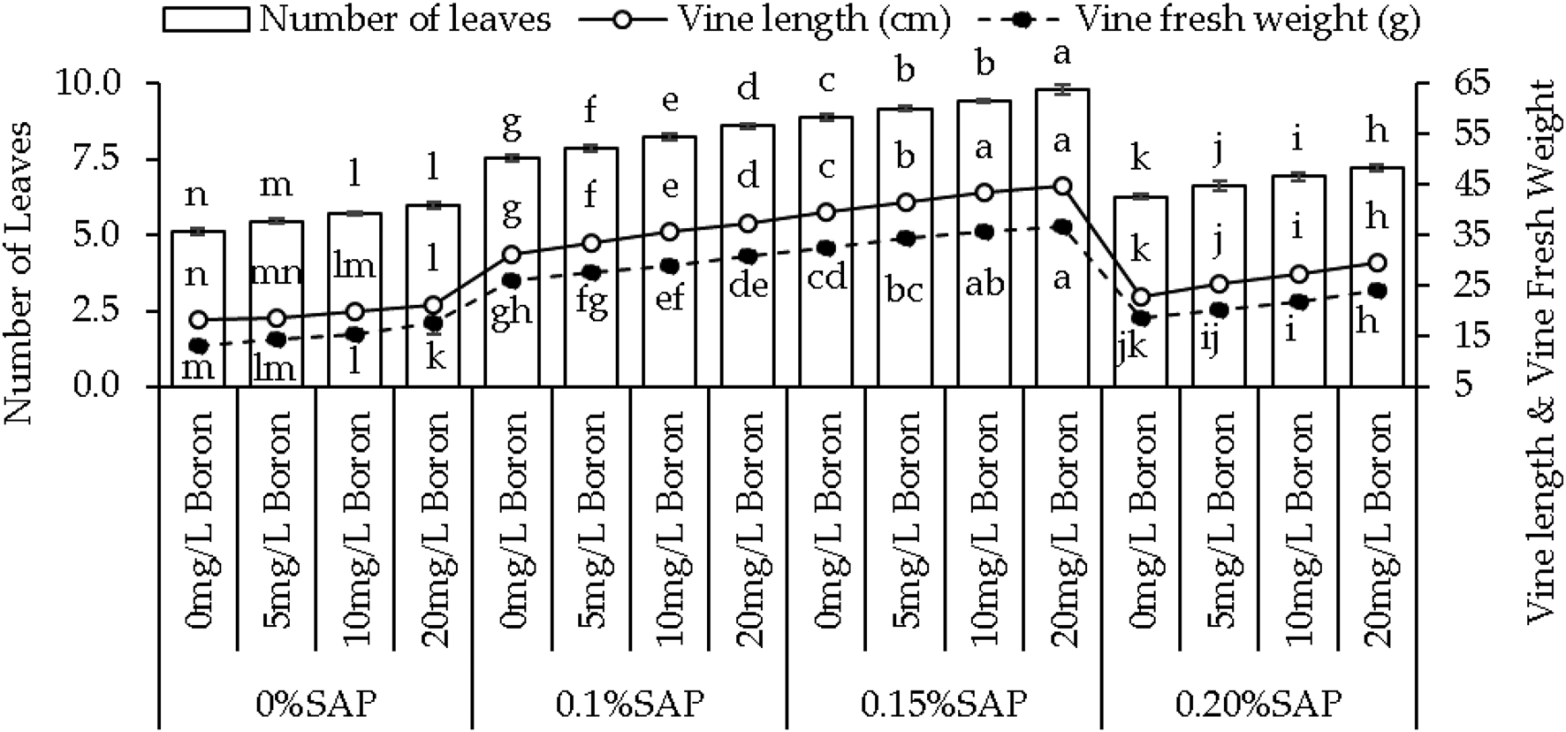
Effect of different concentrations of saponin (SAP) and boron (B) treatments on vine length, vine weight, and number of leaves of sweet potato. Each bar is an average of n = 4 having ± SD showing significant changes at *p* ≤ 0.05 by applying the Tukey test.

For 0% SAP, the addition of 5 mg/L boron (B) resulted in 1.72% increase, while 10 mg/L B and 20 mg/L B showed more significant increases of 8.68% and 16.58%, respectively, compared to control. Moving to 0.1% SAP, the vine length was increased by 7.09%, 14.26%, and 19.91% at 5, 10, and 20 mg/L B over control. At 0.15% SAP, the vine length was enhanced by 4.93%, 9.94%, and 13.12% where 5, 10, and 20 mg/L B were applied over control. In case of 0.20% SAP, 11.04%, 19.98%, and 29.30% improvements were observed in vine length from control at 5, 10, and 20 mg/L B (Fig. 1).

Under 0% SAP, applying 5 mg/L B resulted in 10.26%, 10 mg/L B showed 19.13%, and 20 mg/L B caused 37.05% increase in vine weight than control treatment. In 0.1% SAP, 5.91%, 11.87%, and 19.33% enhancements were noted where 5, 10, and 20 mg/L B were applied over control. Similarly, in 0.15% SAP, the vine weight was increased by 5.40%, 9.33%, and 12.86%, at 5, 10, and 20 mg/L B over control. An improvement of 8.79% in 5 mg/L B, 18.37% in 10 mg/L B, and 30.50% in 20 mg/L B were observed over the control under 0.20% SAP (Fig. 1).

Adding 5 mg/L B resulted in a 6.63% increase in leaf number, while 10 mg/L B and 20 mg/L B led to 12.03% and 17.02% increases than control at 0% SAP. Adding 5 mg/L B + 0.1% SAP led to a 4.24%, while 10 mg/L B + 0.1% SAP and 20 mg/L B + 0.1% SAP caused 9.04% and 13.86% increases, respectively compared to control. In case of 0.15% SAP, 5 mg/L B showed 3.38%, 10 mg/L B and 20 mg/L B concentrations resulted in 6.11% and 10.15% increases in leaf number than control. However, at 0.20% SAP, 5 mg/L B led to a 5.96%, 10 mg/L B and 20 mg/L B showed 10.43% and 15.13% increases, respectively compared to control (Fig. 1).

### Leaf area, root weight, and storage root yield

**Figure 2:**
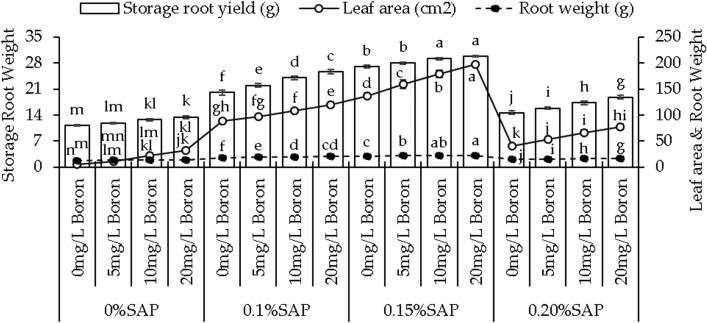
Effect of different concentrations of saponin (SAP) and boron (B) treatments on leaf area, root weight, and storage root yield of sweet potato. Each bar is an average of n = 4 having ± SD showing significant changes at *p* ≤ 0.05 by applying the Tukey test.

Treatment 5 mg/L B showed 128.29%, 10 mg/L B 332.20%, and 20 mg/L B caused 528.29% improvement under 0% SAP. In the case of 0.1% SAP, leaf area was recorded to increase by 9.61%, 21.64%, and 35.76% at 5, 10, and 20 mg/L B, respectively over control. At 0.15% SAP, 16.53%, 30.72%, and 44.00% increase in leaf area was noted where 5, 10, and 20 mg/L B were added compared to control. At 0.20% SAP, leaf area was enhanced 30.75%, 62.05%, and 89.14% in 5, 10, and 20 mg/L B from control (Fig. 2).

Adding boron at 5, 10, and 20 mg/L B for the treatments without SAP led to 4.35%, 7.59%, and 11.27% increases in root weight more than the control. Adding 10% SAP with 5, 10, and 20 mg/L B showed a 4.73%, 10.09%, and 13.23% rise in root weight over the control. At 0.15% SAP, the root weight increased by 3.20%, 5.67%, and 8.31%, with 5, 10, and 20 mg/L B above the control. Adding 0.20% SAP with 5, 10, and 20 mg/L B, the root weight increased by 5.47%, 11.91%, and 17.01% than the control (Fig. 2).

In the case of 0% SAP, adding 5 mg/L B resulted in a 5.85% increase, 10 mg/L B led to a 13.43%, and 20 mg/L B showed a 19.56% increase in storage root yield over the control. With 0.1% SAP, adding 5, 10, and 20 mg/L B resulted in a 9.66%, 20.00%, and 27.91% increase in storage root yield compared to the control. Moving to 0.15% SAP, 5 mg/L B showed a 3.52% increase in storage root yield, 10, and 20 mg/L B exhibit 7.77%, and 10.18% increase above the control. With 0.20% SAP, applying 5, 10, and 20 mg/L B resulted in an 8.23%, 17.75%, and 28.13% rise in storage root yield from the control (Fig. 2).

### Chlorophyll and carotenoid content

**Figure 3:**
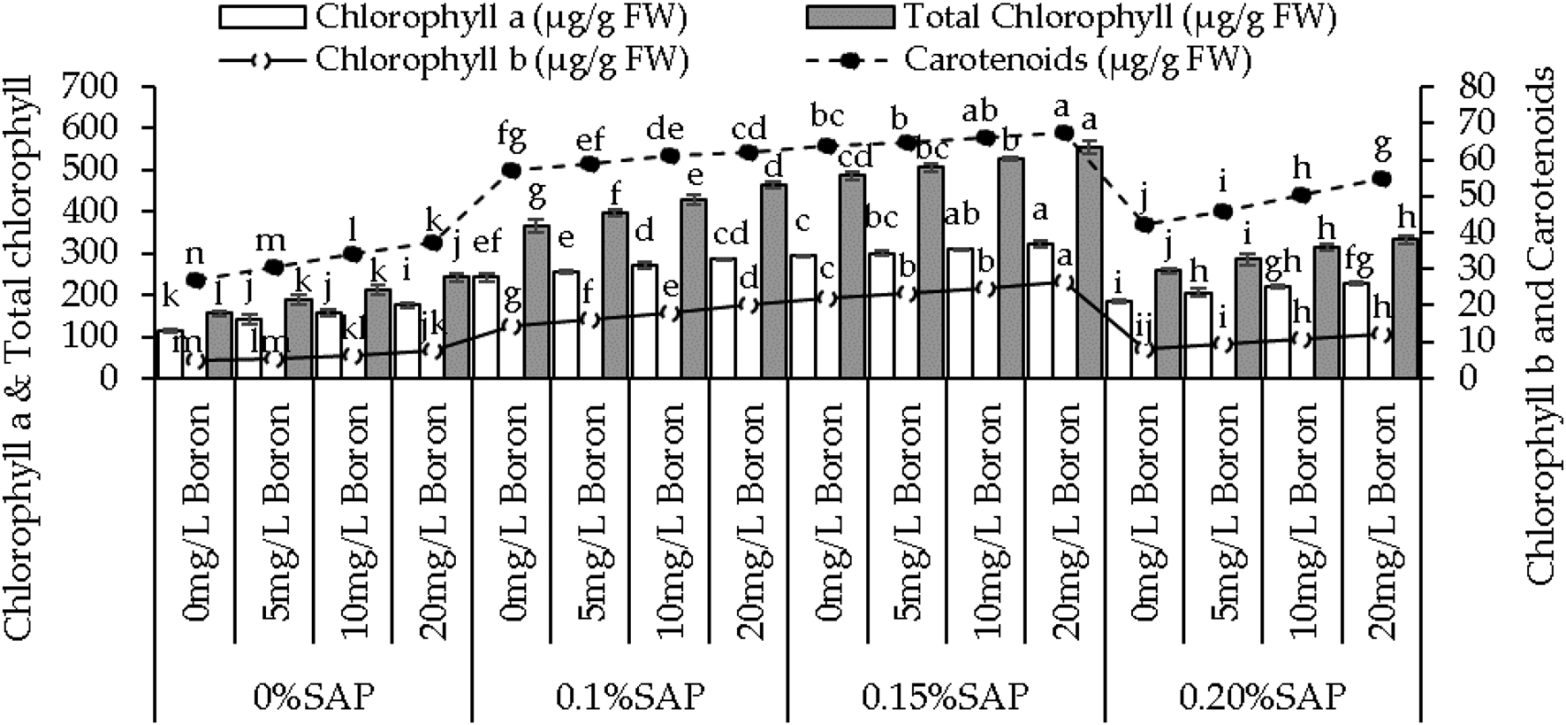
Effect of different concentrations of saponin (SAP) and boron (B) treatments on chlorophyll a, chlorophyll b, total chlorophyll, and carotenoids of sweet potato. Each bar is an average of n = 4 having ± SD showing significant changes at *p* ≤ 0.05 by applying the Tukey test.

Under 0% SAP, adding 5, 10, and 20 mg/L B resulted in a 23.65%, 37.24%, and 53.74% increase in chlorophyll a than the control. In the 0.1% SAP, the chlorophyll a content increased by 5.11% for 5 mg/L B, 11.96% for 10 mg/L B, and 17.73% for 20 mg/L B over the control. Moving to the 0.15% SAP, 2.21%, 5.51%, and 9.84% rise in chlorophyll a was observed with 5, 10, and 20 mg/L B compared to the control. With 0.20% SAP, the chlorophyll a content showed 10.58% increase with 5 mg/L B, 18.25% with 10 mg/L B, and 22.77% with 20 mg/L B from the control (Fig. 3).

At 0% SAP, the introduction of 5 mg/L B showed a 17.64% increase in chlorophyll b, and 10 and 20 mg/L B showed a 34.29% and 59.78% increase than the control. Moving to the 0.1% SAP, the chlorophyll b content increased by 12.85% with 5 mg/L B, 25.80%, and 42.27% increase with 10 mg/L B and 20 mg/L B, respectively, then the control. At 0.15% SAP, the chlorophyll b content showed a 6.86% rise with 5 mg/L B more than the control, 12.48% with 10 mg/L B, and a 20.20% increase with 20 mg/L B. At 0.20% SAP, chlorophyll b content increased by 12.63%, 30.49%, and 45.38% with 5, 10, and 20 mg/L B over the control (Fig. 3).

Adding 5, 10, and 20 mg/L B led to a 22.04%, 36.45%, and 55.35% increase compared to the control under 0% SAP. With 0.1% SAP, applying 5 mg/L B resulted in a 7.74% increase in total chlorophyll content, 10 and 20 mg/L B led to a 16.67% and 26.08% increase than control. Adding 5 mg/L B showed a 4.06% increase, 10 mg/L B showed an 8.27% increase, and 20 mg/L B showed a 13.94% increase over control under 0.15% SAP. With 0.20% SAP, adding 5, 10, and 20 mg/L B resulted in an 11.15%, 21.69%, and 29.11% increase in total chlorophyll content compared to control (Fig. 3).

Applying 5, 10, and 20 mg/L B resulted in a 14.25%, 26.57%, and 38.18% increase in carotenoids than the control under 0% SAP. Adding 0.10% SAP exhibited a 3.24%, 6.70%, and 8.50% increase in carotenoids at 5, 10, and 20 mg/L B concentrations, respectively, over the control. For the 0.15% SAP, carotenoids levels increased by 1.18%, 3.17%, and 5.84%, while the 0.20% SAP increases of 8.87%, 19.25%, and 29.95% at 5, 10, and 20 mg/L B compared to the control (Fig. 3).

### Photosynthetic rate, stomatal conductance, transpiration rate, and Fv/Fm

**Figure 4:**
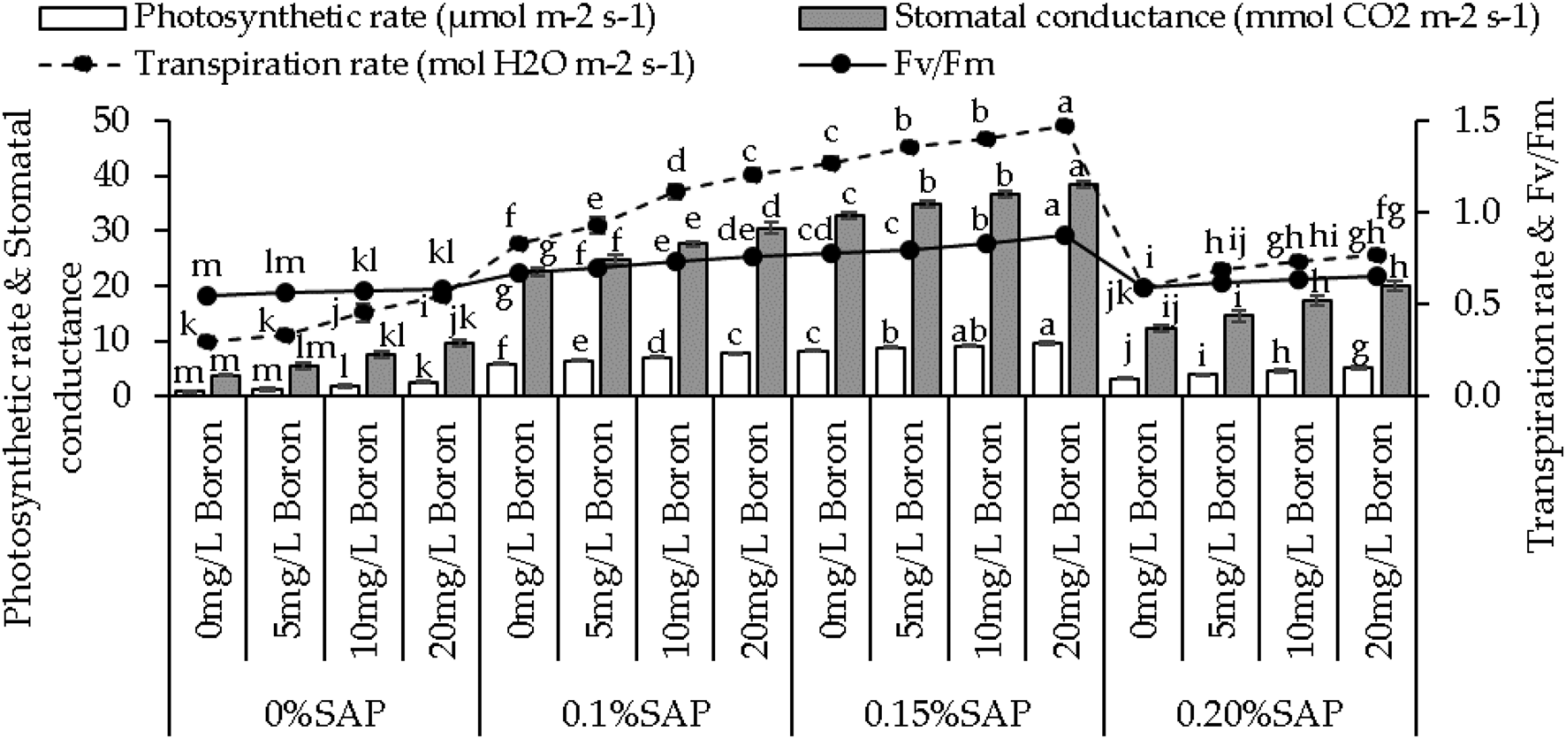
Effect of different concentrations of saponin (SAP) and boron (B) treatments on photosynthetic rate, stomatal conductance, transpiration rate, and Fv/Fm of sweet potato. Each bar is an average of n = 4 having ± SD showing significant changes at *p* ≤ 0.05 by applying the Tukey test.

With 0% SAP, the photosynthetic rate showed 32.74%, 99.66%, and 184.23% increase with 5, 10, and 20 mg/L B compared to the control. At 0.1% SAP, adding 5 mg/L B resulted in a 10.39% increase in photosynthetic rates, 10 and 20 mg/L B showed a 20.64% and 31.11% increase to the control. Adding 5, 10, and 20 mg/L B showed 7.52%, 11.54%, and 17.69% increase in photosynthetic rate over the control under 0.15% SAP. with 0.20% SAP, adding 5, 10, and 20 mg/L B resulted in a 20.71% increase in photosynthetic rate, 10 and 20 mg/L B showed a 41.59% and 63.07% rise from the control (Fig. 4).

In the case of stomatal conductance, at 0% SAP, the addition of 5, 10, and 20 mg/L B led to 48.10%, 99.80%, and 154.24%, while with 0.1% SAP showed 9.69%, 22.25%, and 34.87% increases. Under 0.15% SAP, the stomatal conductance was increased by 6.63%, 11.86%, and 17.59% by adding 5, 10, and 20 mg/L B, and with 0.20% SAP resulted in 19.45%, 42.17%, and 64.86% increase (Fig. 4).

For 0% SAP, an increase in transpiration rate by 13.46% with 5 mg/L B, 52.82% with 10 mg/L B, and 82.67% with 20 mg/L B than the control. Transpiration rates increased by 11.27% with 5 mg/L B, 33.24% with 10 mg/L B, and 44.25% with 20 mg/L B related to the control under 0.1% SAP. For 0.15% SAP, the transpiration rates increased by 6.53%, 10.00%, and 16.03%, and with 0.20% SAP showed 17.30%, 23.77%, and 30.08% with 5, 10, and 20 mg/L B compared to the control (Fig. 4).

Under 0% SAP, Fv/Fm ratio showed a 3.22%, 5.25%, and 6.86% increase, and with 0.1% SAP resulted in a 4.13%, 9.63%, and 12.64% increase more than the control. Adding 5 mg/L B with 0.15% SAP led to a 2.15% increase in Fv/Fm, 10 mg/L B resulted in a 6.28% increase, and 20 mg/L B showed a 12.25% increase from the control. With 0.20% SAP, adding 5 mg/L B resulted in a 3.32% increase in Fv/Fm, 10 mg/L B led to a 7.14% increase, and 20 mg/L B showed a 9.29% increase over the control (Fig. 4).

### Photon yield of PSII and soluble protein

**Figure 5:**
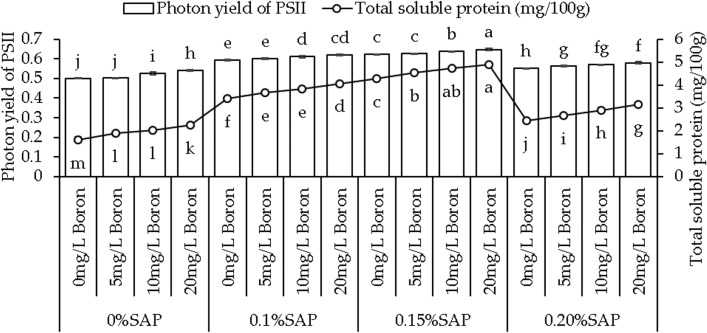
Effect of different saponin (SAP) and boron (B) treatment concentrations on sweet potato photon yield of PSII and total soluble protein. Each bar is an average of n = 4 having ± SD showing significant changes at *p* ≤ 0.05 by applying the Tukey test.

Photon yield of PSII increased by 0.60%, 4.65%, and 8.14% with 5, 10, and 20 mg/L B concentrations with 0% SAP than the control. Applying 0.1% SAP, the photon yields significantly increased by 1.39%, 3.12%, and 4.52%, and at 0.15% SAP, showed 0.52%, 2.17%, and 3.85% increase with 5, 10, and 20 mg/L B than the control. With 0.20% SAP, the photon yield increased by 2.18%, 3.68%, and 5.27% at 5, 10, and 20 mg/L B concentrations, respectively, over control (Fig. 5).

Under 0% SAP, adding 5, 10, and 20 mg/L B total protein contents showed a 17.26%, 25.97%, and 39.66% increase, respectively, from the control. With 0.1% SAP, adding 5, 10, and 20 mg/L B the total soluble protein content increased by 7.66%, 12.81%, and 19.81%, and with 0.15% SAP, showed 6.21%, 10.60%, and 14.81% increase compared to the control. However, at 0.20% SAP, the protein content showed increases of 8.67%, 18.57%, and 28.88% at the 5 mg/L B, 10 mg/L B, and 20 mg/L B concentrations, respectively, from the control (Fig. 5).

### Proline content, MDA, DPPH, and ABTS activity

**Figure 6:**
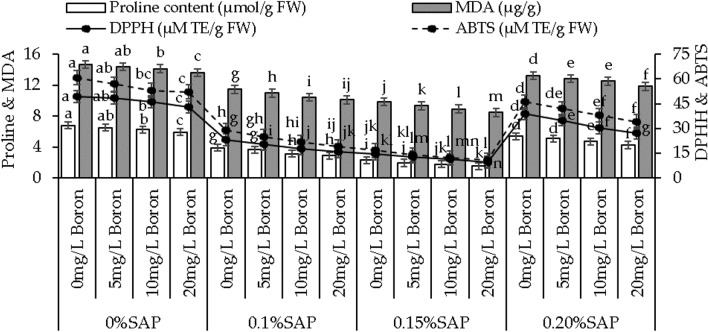
Effect of different concentrations of saponin (SAP) and boron (B) treatments on proline content, malondialdehyde (MDA), DPPH, and ABTS of sweet potato. Each bar is an average of n = 4 having ± SD showing significant changes at p ≤ 0.05 by applying the Tukey test.

In 0% SAP, adding 5, 10, and 20 mg/L B, caused 4.42%, 8.62%, and 14.73% decrease in proline than the control. With 0.1% SAP proline content decreased by 6.90%, 24.49%, and 35.49%, and with 0.15% SAP resulted in 19.68%, 31.96%, and 51.92% decrease was observed with 5, 10, and 20 mg/L B than the control. At 0.20% SAP, the addition of 5 mg/L B resulted in a 5.74% decrease in proline content over the control, a 15.16% decrease with 10 mg/L B, and 26.83% with 20 mg/L B (Fig. 6).

At 0% SAP, adding 5 mg/L B resulted in a 1.95% decrease in MDA activity, while 10 mg/L B and 20 mg/L B led to 3.78% and 7.59% decrease, respectively, then the control. For 0.1% SAP concentration, adding boron at 5, 10, and 20 mg/L B led to a 4.42%, 10.07%, and 13.36% decrease in MDA activity over the control. Compared to the control, adding 0.15% SAP with 5, 10, and 20 mg/L B resulted in a 5.51%, 10.30%, and 16.44% decrease in MDA activity. With 0.20% SAP, adding 5, 10, and 20 mg/L B showed a 3.29%, 5.74%, and 11.76% decrease in MDA activity compared to the control (Fig. 6).

Appling 5, 10, and 20 mg/L B with 0% SAP resulted in a significant 1.80%, 6.51%, and 14.58% decrease in DPPH activity compared to the control. Adding 0.1%, the DPPH activity decreased by 14.93%, 31.29%, and 45.24%, and with 0.15% SAP, led to a 13.28%, 29.88%, and 50.77% decrease by the addition of 5, 10, and 20 mg/L B from the control. When 0.20% SAP was applied with 5, 10, and 20 mg/L B resulted in an 11.16%, 28.31%, and 43.22% decrease in DPPH activity over the control (Fig. 6).

For 0% SAP, adding 5 mg/L B resulted in a 6.70% decrease in ABTS activity, 10 mg/L B exhibited a 14.57% decrease, and 20 mg/L B 16.55% decrease compared to the control. In 0.1% SAP, adding 5, 10, and 20 mg/L B showed a 16.06%, 33.21%, and 52.21% decrease in ABTS activity, while 0.15% SAP exhibited a significant 18.87%, 37.06%, and 55.77% decrease over the control. With 0.20% SAP, applying 5 mg/L B showed a 9.77% decrease, 10 mg/L B exhibited a 21.14% decrease, and 20 mg/L B resulted in a 35.36% decrease in ABTS activity than the control (Fig. 6).

### Convex hull and hierarchical cluster analysis

**Figure 7:**
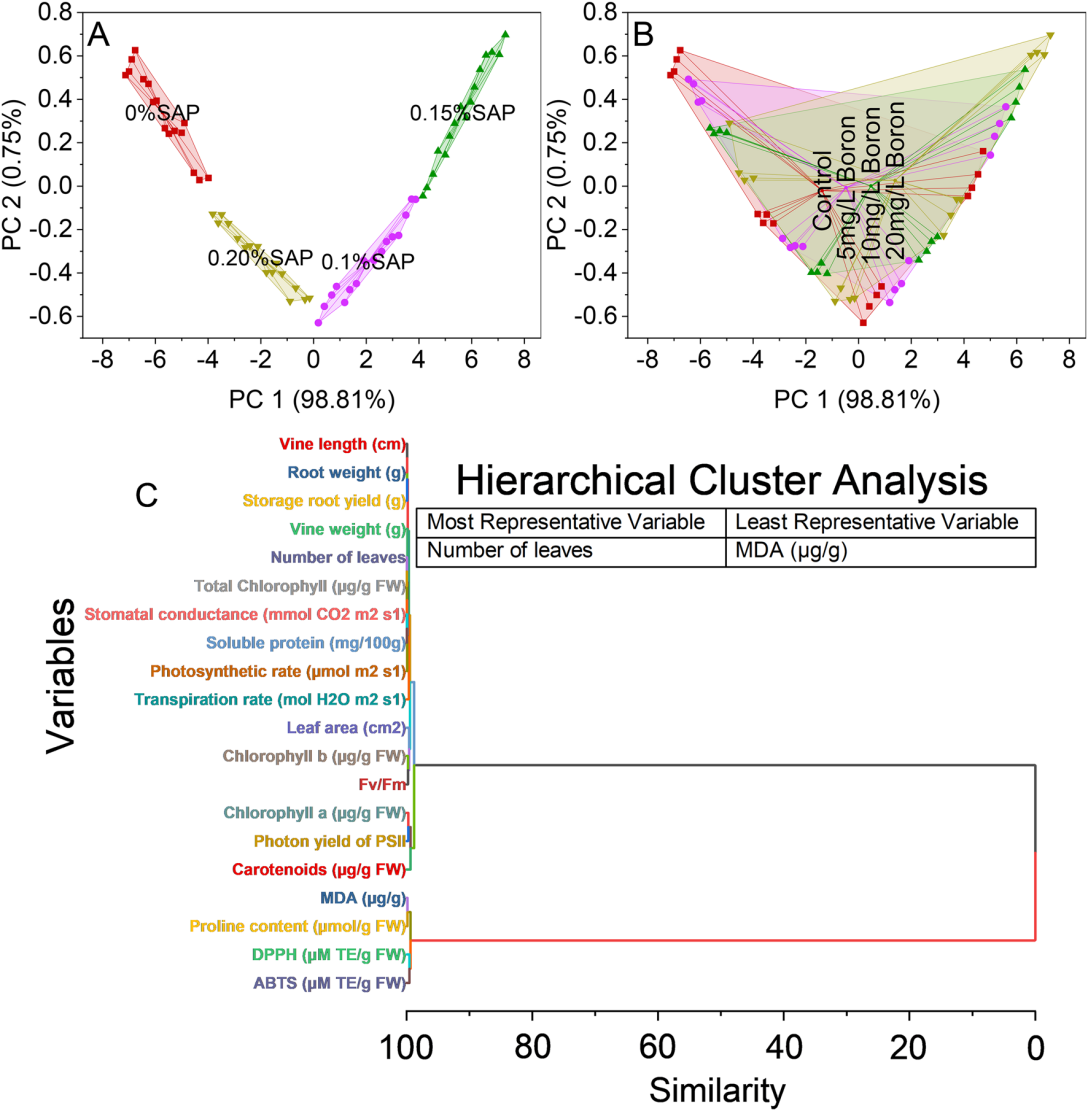
Cluster plot convex hull for saponin (SA) levels (**A**), boron (B) levels (**B**), and hierarchical cluster plot (**C**) for studied attributes.

The convex hull analysis was conducted on a dataset of coordinates represented in a two-dimensional space (PC 1 and PC 2) and labeled according to different percentages of SAP (SAP). The analysis indicates that at 98.81% accuracy along PC 1 and 0.75% accuracy along PC 2, the data points within the categories of 0% SAP, 0.1% SAP, 0.15% SAP, and 0.20% SAP form distinct convex shapes or boundaries when plotted on a graph (Fig. 7A).

The control group likely represents a standard or baseline condition, comprising data points with scores within the PC 1 and PC 2 ranges. Meanwhile, the 5 mg/L B, 10 mg/L B, and 20 mg/L B treatments signify varying experimental conditions or interventions, showing different patterns in the dataset (Fig. 7B).

The first cluster amalgamates variables associated with physiological processes, including photosynthetic and transpiration rates, leaf area, and chlorophyll content, suggesting an interconnectedness in plant function. Another cluster identifies variables linked to antioxidant capabilities, such as DPPH and ABTS measurements, implying a shared characteristic of the plant’s antioxidative properties. Moreover, a distinct cluster forms around pigmentation-related variables like chlorophylls, carotenoids, and photon yield of PSII, potentially indicating a cohesive group related to plant pigmentation and photosynthetic activity. Additionally, the analysis reveals a cluster involving variables like MDA and proline content, likely associated with stress responses or cellular protection mechanisms within the plant (Fig. 7C).

### Pearson correlation analysis

**Figure 8:**
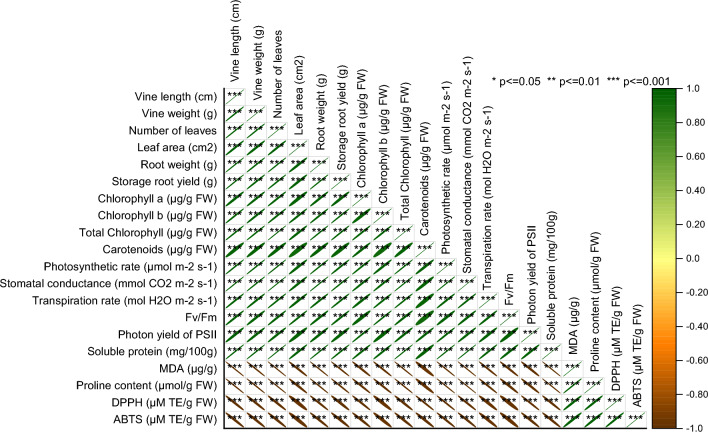
Pearson correlation for studied attributes.

Numerous strong positive correlations are evident among multiple parameters, including vine length, vine weight, number of leaves, leaf area, root weight, chlorophyll concentrations, photosynthetic rate, stomatal conductance, and soluble protein. These correlations, approaching or reaching values close to 1, imply a direct relationship or proportionality between these factors, suggesting that changes or variations in one parameter are likely reflected in others within this group. Additionally, moderate to high positive correlations exist among variables like transpiration rate, Fv/Fm, and photon yield of PSII, indicating their interconnectedness and potential mutual influence. Conversely, strong negative correlations are observed among measures such as MDA, proline content, DPPH, and ABTS, signaling an inverse relationship or potential opposing impacts between these variables and the rest of the parameters (Fig. 8).

## Discussion

Boron is an essential micronutrient for plant growth, influencing various physiological processes. Boron is a vital micronutrient for plant growth, influencing cell wall formation, carbohydrate metabolism, nucleic acid synthesis, cell membrane stabilization, cell division, and elongation, enhancing plant development^30^. This micronutrient often acts as a cofactor for various enzymes involved in cell wall synthesis, thereby influencing elongation processes and contributing to increased vine length^31^, as shown in (Fig. 1A). Moreover, boron plays a role in sugar transport within the plant, impacting sink-source relationships. This involvement in sugar translocation can potentially contribute to increased vine weight, as shown in (Fig. 1B), as sugars are essential to produce biomass and storage compounds^32^. SAP are bio-stimulants that enhance plant growth by improving nutrient uptake, root growth, and stress tolerance^33^. They facilitate soil nutrient absorption, including boron, potentially increasing vine growth parameters. When combined with boron, SAP enhances boron’s impact on cellular processes^34^. This combined effect may stimulate cell division, elongation, and metabolic activities, leading to increased vine length and leaf number, as shown in (Fig. 1A,C). The interaction between SAP and boron may trigger growth-related gene expression, activating specific genes and improving vine growth metrics. Leaf area expansion likely results from the combined impact of SAP, known for altering cell membrane permeability and aiding nutrient uptake, and boron, crucial for cell wall formation^35^. Their collaboration enhances cell division and expansion, contributing to increased leaf area. In root development, SAP’s influence on root exudation and microbial communities and boron’s role in cell wall structure likely synergize to promote root growth and branching^36^. This interaction improves nutrient uptake efficiency and structural development. Additionally, the combined effect of SAP and boron on storage root yield may arise from enhanced photosynthetic efficiency due to SAP and optimized carbohydrate translocation facilitated by boron. This collaborative influence optimizes assimilate allocation, boosting storage root yield shown in (Fig. 2C). The mechanisms involve improved nutrient uptake, fortified cell walls, efficient nutrient translocation, and heightened photosynthetic efficiency. SAP may act as signaling molecules affecting growth and stress response pathways, while boron supports enzymatic reactions and structural components crucial for these processes^37^. The increase in chlorophyll levels, particularly chlorophyll a and b, owes credit to boron’s pivotal role in chlorophyll synthesis^38^. Boron aids chlorophyll formation by supporting chloroplast membrane stability, which is crucial for assembling photosystems and electron transport in photosynthesis^39^. It also influences enzymes involved in chlorophyll biosynthesis, enhancing precursor conversion into chlorophyll. The dose-dependent response to boron levels underscores its importance in optimizing chlorophyll production. Furthermore, boron’s impact on chloroplasts indirectly affects carotenoid accumulation, which is pivotal as a protective pigment in photosystems^40^. Boron’s positive influence on chlorophyll likely fosters an environment for increased carotenoid synthesis, mutually enhancing light capture and energy utilization in photosynthesis^41^. The interplay between SAP and boron observed in this study might involve SAP aiding boron uptake or influencing plant processes, indirectly shaping boron’s availability or metabolic effects within plant tissues. Boron’s pivotal role in photosynthesis involves optimizing enzymatic reactions within chloroplasts, enhancing chlorophyll synthesis, and regulating carbon fixation processes, which substantially increases photosynthetic rates^42^. Its influence extends to stomatal regulation, impacting water movement and nutrient transport, which are crucial for overall plant growth. SAP complement these effects by potentially improving membrane integrity and nutrient uptake^43^. The rise in Fv/Fm ratios signifies improved photosystem efficiency, attributed to boron’s optimization of electron transport and chlorophyll protection, with SAP potentially enhancing these benefits. Boron influence on PSII stability contributes to increased photon yield, potentially supported by SAP’ role in PSII protection against oxidative stress^44^. The fluctuations in soluble protein levels may arise from intricate interactions among boron, SAP, and protein metabolism pathways, influencing protein synthesis and turnover processes. Boron supplementation and SAP presence affect plant growth, stress responses, and proline content. Boron decreases proline content, suggesting osmotic balance regulation and reducing proline accumulation in response to stress^45^. According to the hierarchical cluster analysis, MDA is the least parameter to increase plant growth, as shown in (Fig. 7C). The decrease in MDA, a marker of lipid peroxidation and oxidative stress, with boron addition hints at its role in mitigating oxidative damage. Boron might participate in antioxidant enzymatic systems or directly scavenge reactive oxygen species (ROS), reducing lipid peroxidation and oxidative stress levels^46^. This could signify a protective mechanism against oxidative damage induced by stressors. Regarding growth enhancement mechanisms, the observed alterations in proline content, MDA activity, and antioxidant capacities could indirectly contribute to improved growth. Reduced proline levels may signify an optimized stress response, allowing plants to allocate resources toward growth rather than stress adaptation. Decreased lipid peroxidation and enhanced antioxidant activities might contribute to a less stressful cellular environment, enabling plants to allocate energy and resources toward growth processes.

## Conclusion

In conclusion, the combination of 0.15% SAP and 20 mg/L B emerged as the most effective treatment in mitigating salinity stress for sweet potatoes. This combination notably improved various growth parameters and physiological aspects, highlighting its potential as a recommended amendment for addressing salinity-related issues in this crop. The study suggests a potential solution to alleviate salinity-induced limitations on sweet potato cultivation, potentially revolutionizing crop management strategies and contributing to food security. Future research should explore this treatment’s long-term effects and scalability under different environmental conditions.

## Data Availability

All data generated or analysed during this study are included in this published article.
